# Supporting detection of hostile intentions: automated assistance in a dynamic decision-making context

**DOI:** 10.1186/s41235-023-00519-5

**Published:** 2023-11-19

**Authors:** Colleen E. Patton, Christopher D. Wickens, C. A. P. Smith, Kayla M. Noble, Benjamin A. Clegg

**Affiliations:** 1 Department of Psychology, Colorado State University, Fort Collins, USA; 2 Montana State University, Bozeman, MT 59717 USA

**Keywords:** Dynamic decision making, Automation, Trust, Transparency

## Abstract

In a dynamic decision-making task simulating basic ship movements, participants attempted, through a series of actions, to elicit and identify which one of six other ships was exhibiting either of two hostile behaviors. A high-performing, although imperfect, automated attention aid was introduced. It visually highlighted the ship categorized by an algorithm as the most likely to be hostile. Half of participants also received automation transparency in the form of a statement about why the hostile ship was highlighted. Results indicated that while the aid’s advice was often complied with and hence led to higher accuracy with a shorter response time, detection was still suboptimal. Additionally, transparency had limited impacts on all aspects of performance. Implications for detection of hostile intentions and the challenges of supporting dynamic decision making are discussed.

## Significance statement

The current research investigates human–automation teaming and automation transparency in a dynamic decision context. Dynamic decision making (Gonzales et al., 2017) is based upon real-world decisions that require evidence accumulation and shorter decision cycles within a larger decision. This is often seen in medical and military contexts, as well as when predictions are made about future states. These decisions are difficult for humans to make accurately, which creates a place to introduce automated aids. This research addresses this issue in the context of a visual search and pattern recognition task that mimics Naval ship detection from a radar screen. The current findings suggest that while automated attentional aids are useful in dynamic decision-making contexts, transparency does not improve performance in these complex environments. This creates a foundation for application to real-world instances of dynamic decision making.

## Introduction

Along with reducing human mental workload and the costs of operation, one of many generic functions of introducing automation or artificial intelligence to a human-centric environment is to improve the performance of the human–automation team (HAT). Automation can be presented in many forms, but the focus of the current paper is on automation decision/diagnostic support and its integration into dynamic decision making.

### Dynamic decision making

Dynamic decision making (DDM), as reviewed by Gonzales et al. (2017), see also Edwards (1962), is based upon the observations that decision tasks are not often static, simple decisions but, instead, they have dynamic complexity that emerges from various choices made over time and their effects on subsequent choices. In many dynamic decision tasks, at each choice, a decision is made whether to stop, or to continue analyzing or sampling each new option and/or acquire additional evidence. To decide whether they should continue analyzing or sampling, a person looks for the point when the value of the current option either exceeds the value of a future option, or if it meets a certain threshold.

The unique features of DDM as opposed to static decision making are that the decider will encounter multiple packages of data over time, often making a temporary decision or situation assessment after each data point and then revising it in the face of new data. Such an evidence accumulation process would readily describe the decision process of a healthcare professional diagnosing a patient condition as symptoms are encountered over time. Often the actual decision made at time T1 will impose an action (for example a treatment administered to the patient), whose outcome then provides data for a decision or diagnosis revision at time T2. Heavy cognitive demands tend to be imposed by DDM, in comparison with the general situation with static decision making. In particular, working memory is involved in retaining information about previous states and when evaluating the outcome of present decisions and diagnoses (Herdener et al., 2019). Such working memory demands of evidence accumulation provide an added source of cognitive load not present in comparable static, one-shot decision making.

It is noteworthy, and relevant to the current research, that studies of DDM typically involve two levels of learning (Gonzales et al., 2017). On a lower level, as evidence is accumulated there is a form of short-term learning. At a higher level, learning can occur across trials or final decisions, such that it is closer to cognitive skill development. We examine both forms here.

Pairing the process down into simple repeated decisions within a laboratory setting allows exploration of decision making and how automation support may impact that process, and these types of dynamic decision tasks are common in the real world. Yet, to our knowledge, only one previous study has investigated automation assistance in a dynamic decision-making context (Kleinmuntz & Thomas, 1987), and transparency in the DDM context has yet to be explored. This is not to say that human–automation teaming in real-world simulations lacks exploration, as this area is abundant (e.g., Chen & Barnes, 2012; Chien et al., 2018; Hutchinson et al., 2022; O’Neill et al., 2022; Sargent et al., 2023; Strickland et al., 2023) but rather, the interest here is on the impact of automation in evidence accumulation (i.e., dynamic decision making) settings. The intersection of three elements, *automation support* for *dynamic decision making*, and how both the process and product of dynamic decision making may be influenced by *automation transparency* represents the unique contribution of the current research.

### Automation support

Decision-making performance can be improved through the use of a diagnostic support system; however, in such evaluations, human–automation team (HAT) performance must be evaluated against two benchmarks: performance of the human alone (unaided by automation) and performance of the automation alone. A general finding is that HAT performance typically lies somewhere in between the two benchmarks, and how close it falls to one or the other depends on an array of factors. For example, in a task that is difficult for an unaided human but has support from a highly reliable aid, the HAT performance would move closer to the automation’s performance level, but typically without actually reaching that optimal level (Bartlett & McCarley, 2017, 2020; Boskemper et al, 2021).

Thus, a question that arises in this instance is that if the aid is superior, and humans wish to optimize HAT performance, why do humans not entirely depend upon the aid, always following aid guidance? One answer may be the differences between humans’ intrinsic trust in the aid (Lee & See, 2004). In particular, humans themselves show a wide range of what is termed dispositional trust (Hoff & Bashir, 2015). This may lead to a general trend toward under-trust for some human users who then depend on automation less than is optimal. In contrast, other people may demonstrate near total trust, and in some cases, poorly calibrated trust can lead to poorly calibrated dependence or compliance. When considering over-dependence, this can lead to the *automation bias* of not paying any attention to the raw data, and hence remaining totally out of the loop (Mosier et al., 1998; Parasuraman & Manzey, 2010).

The automation bias can produce two consequences: First, poor performance on the infrequent occasions when imperfect automation is incorrect or unexpectedly fails, a shortcoming that can often be attributed to a loss of situation awareness (Endsley, 2017; Kaber & Endsley, 1997; Trapsilawati et al., 2021); second, failing to learn or forgetting how to do the task that is supported by automation, and hence doing poorly when it is withdrawn. The latter is a phenomenon sometimes referred to as *deskilling* (Casner et al., 2014; see also Bainbridge, 1983). While a case can be made that an operator using or depending upon automation excessively can lose this skill; an alternative case can be made that the reduced workload availed by automation when it is in use could allow the user to allocate more resources to learning and understanding the decision and diagnostic strategies underlying task performance and hence be actually better at the task following automation withdrawal (see Gutzwiller et al., 2013). We examine these issues in the current study.

### Transparency

A potential solution to reducing deskilling or increasing learning in a task is fostering better situation awareness when automation unexpectedly fails and fostering better learning of the decision/diagnostic task. This may be supported by transparency. Automation transparency is a concept that has number of different operational definitions (e.g., Dzindolet et al., 2003; Hoff & Bashir, 2015; Schneiderman, 2022; Seong & Bisantz, 2008; van de Merwe et al., 2022), but at its broadest level, it is a property of automation that enables the human user to understand what the automation is doing and/or why it is doing it. Transparency types can be categorized in a number of ways; however, one widely accepted way is based on the information provided to the user about the automation. Bhaskara et al. (2020) lay out “low”, “medium” and “high” levels of transparency, corresponding to the type of information the user sees (see Mercado et al., 2016 for examples of all three levels). Low transparency contains basic information about the system—its intent, a baseline plan, etc. The medium level of transparency provides an explanation of the reasoning behind the automation decision advice—how the algorithm works, or why it arrived at the particular recommendation that it did. The high level of transparency provides an estimation of the confidence with which automation offers its advice (Kunze et al., 2019).

While transparency is not required to include reasoning or confidence, a review of the literature suggests that any lesser transparency manipulation (i.e., only the outline of a plan) does not generally increase performance metrics (Bhaskara et al., 2020). Two recent reviews of the transparency literature (Bhaskara et al., 2020; van de Merwe et al., 2022) and a meta-analysis (Sargent et al., 2023) indicate that with these reasoning manipulations, HAT performance typically increased, particularly for instances where the human was responding to automation suggestions for decision making. While the transparency literature reveals that its incorporation improves the performance of the HAT most of the time (e.g., Chen & Barnes, 2012; Seppelt & Lee, 2019), such improvement is not universally observed (e.g., Guznov et al., 2020; Pharmer et al., 2021; Wright et al., 2016) and may occasionally even inhibit it (Zhang et al., 2022). Thus, the positive relationship between increased transparency and improved performance is not guaranteed.

One reason why transparency at any level may fail to benefit is because users simply do not attend to it. Explanations may be complex, and if rendered in printed text, may be hard to read, thereby competing with the resources needed to both process the raw data and to learn the task (e.g., Kunze et al., 2019). One might thus speculate that as decision tasks become increasingly difficult and therefore resource demanding, while automation itself can help, the added benefits of transparency for that automation may fail to emerge, as they are offset by the cost of divided attention (Tatasciore et al., 2023).

### Trust in automation

One area in which transparency appears to clearly have a positive effect on the human–automation team is in helping a user calibrate their trust in an automated system according to its degree of reliability (Hoff & Bashir, 2015; Sargent et al., 2023; Vorm & Combs, 2022). It follows that transparency generally increases performance as well, because if trust is properly calibrated and the automated system is more accurate than the human, the two should be able to work together to produce better accuracy. However, it is important to note that trust and dependence are not always directly related. People’s trust in automation may not predict their ability to detect automation failures (Merritt et al., 2013), and changes in accuracy or reliability of an automated aid may not result in a corresponding change in trust of or dependence on that aid (Korbelak et al., 2018; Hutchinson et al., 2022).

Trust in the automation is also thought to be influenced by the task load (Chien et al., 2018; Zhang & Yang, 2017). Specifically, there is evidence that trust increases with task difficulty (Sato et al., 2020). In a dynamic decision-making task, where the task load is high, it would be expected that trust also be high, but whether this leads to overreliance on the automation in these contexts is not yet known. This issue is examined in the current experiment.

### Current study

One way in which all of this research on transparency, trust, and automation dependence is lacking in is in understanding the impacts of transparency on the decision-making *process* rather than the outcome. It is not clear how transparency impacts cognitive mechanisms in a way that leads to better performance. This may be, in part, because conclusions thus far tend to focus on situations in which a single decision is made from a set amount of evidence. Yet, there are environments and situations where evidence can accumulate over time, such as evaluating the changing threat of a spreading forest fire, a military adversary on the move, or the progression of a disease (Kleinmuntz & Thomas, 1987). These issues are addressed under the dynamic decision-making approach (Gonzales et al., 2017). This environment may allow transparency to be investigated in a different role, influencing not just decision outcomes but also the *process* by which dynamic decisions are made and unfold over time. To examine these issues, we focused on a dynamic decision task involving the identification of hostile intent (Riviero et al., 2017). To examine this, we built upon our prior research in which participants sought to identify a hostile ship within a set of moving ships and diagnose which of two hostile behavior types was shown (Patton et al., 2021a; 2021b, 2022). A shadowing ship stalked the participant’s ship through moves parallel to the own ship, and a hunting ship gradually approached the user ship. This environment captures prototypical features of Naval combat information center displays such as the Aegis (Smith et al., 2004) and the task also mimics, in some ways, a real Naval task of a ship’s intelligence officer monitoring radar tracks. Introducing variability into the ship movements can reflect natural variations of vessel movement due to tides, winds, or other mission-related maneuvers. Deviations from normal paths and close approaches are both real ship movements that can indicate hostility (Lane et al., 2010). While this is a simplified laboratory paradigm, the intention was that the cognitive and environmental variables adequately represent a prototypical real-world detection scenario.

In the current paradigm, the behavioral diagnosis is accomplished by maneuvering the ownship in a series of steps or “moves” in any of four cardinal directions the participant chooses, while observing the behavior of all the ships to see which is responsive to the ownship moves (identification of suspect ship) and the nature of that response (approaching, as in hunting, vs parallel, as in shadowing). The freedom to end a trial when the participant feels they have accumulated enough evidence to accurately identify the hostile ship allows the number of moves that a participant uses to be a proxy for the meta-cognitive process of deciding when the threshold of necessary evidence has been met—a hallmark metric of dynamic decision making. By this cyclical process of decision (how to move) and observation (of movement of other ships), users accumulate evidence until they are confident of the diagnosis, respond with the identity of the suspected ship, and terminate the trial. Thus, there are heavy spatial working memory demands of keeping track of movement of all ships, in order to then discern which of these, over several consecutive moves, have consistently responded in a manner contingent upon the direction of the ownship’s movement (Patton et al., 2022). Previous research indicates the task is difficult for an unaided human, with accuracy around 50% (chance of 8% [6 ships × 2 behaviors]; Patton et al., 2021a; 2021b, 2022).

The current study introduced an attention cuing automated aid to attempt to achieve improvement on detection and diagnostic performance by gathering and integrating the movement from each ship to assess the extent to which that movement was consistent with one hostile behavior or the other. It then highlighted the most likely target. The goal of the current study was thus twofold. First, to understand the degree of assistance the imperfect automation decision aid could provide to the user in making the final diagnostic decision as to the hostile element and the manner in which performance was supported. Previous literature would suggest that the imperfect aid would improve performance, as its overall accuracy is better than the unaided human (Bartlett & McCarley, 2017, 2020; Boskemper et al., 2021), but the extent to which it benefits an evidence accumulation task is of interest. Second, whether this support could itself be improved further by introducing automation transparency. The form of transparency that we employed here was a reasoning explanation for why the automated diagnostic aid recommended the ship that it did as hostile, based on its recent history of contingent movements. This recommendation was placed on the screen, after each participant-initiated move.

Four hypotheses were put forth:

1. Because the aid’s accuracy is so much higher than the unaided participant, it is predicted that, in line with previous literature (Boskamper et al., 2021; Kleinmuntz & Thomas, 1987), participant accuracy on this dynamic decision-making task will improve with the aid.
2. The availability of the automated aid should reduce the cognitive processing for each decision, in part by offloading working memory, and hence provide an opportunity for the participant to learn the effective strategies of performing the task. To the extent that this opportunity is used, this in turn should support better performance if the aid is removed, as occurs at the end of the current task.
3. The transparency manipulation will improve performance further because it is providing additional information (reasoning, i.e., Bhaskara et al., 2020) that can be used by the participant to better calibrate their trust and use of the aid.
4. Transparency will change the way users make decisions. This may be seen in differences between the transparent and non-transparent conditions in number of steps used across trials, compliance rates, or differences in accuracy when the aid is taken away.

## Methods

### Participants

The current research was approved by the Colorado State University Institutional Review Board. Data were collected from 128 people on Prolific, all of whom were located within the USA and gave informed consent prior to starting the experiment. Demographic information was not collected. Two participants were removed due to evidence of inattention, based on few moves per trial (less than 7 out of 35), and exceptionally poor performance (lower than chance).

### Task

**Fig. 1:**
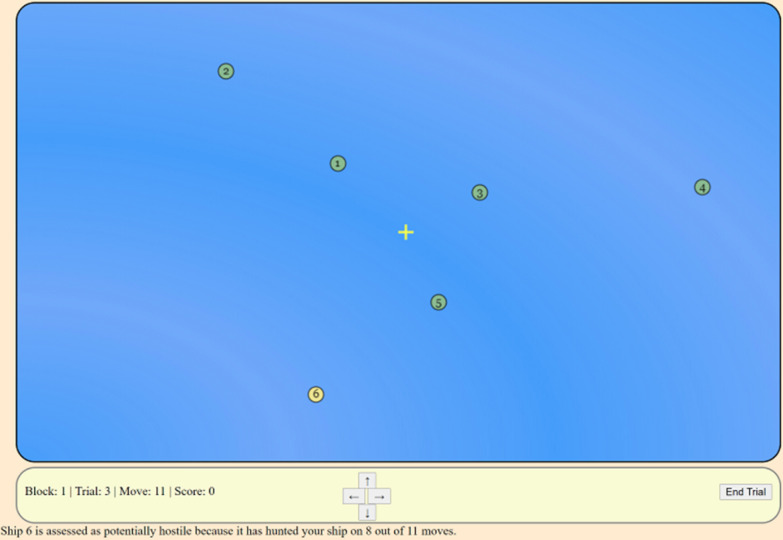
Screen exhibiting the experimental paradigm

The experiment featured a modified version of the task from Patton et al. (2021a). Participants viewed a computer screen (Fig. 1) containing a yellow cross indicating their ship’s position (which they could control), and six green circles with unique numbers which represented other ships and were controlled by a software application. An example of this task can be found here: https://osf.io/3hd9t/.

On each trial, the starting location of all ships was randomly assigned, with the participant’s ship starting close to the middle of the screen. The participants could move their ship by clicking the arrow keys on the bottom of the screen; participants could click no faster than once per second. The user ship could move left, right, up, or down, and each time the user ship moved, the computer-controlled ships also moved in a pre-programmed direction. Thus, all ships moved at the same time, with at least a one second delay between movements.

On each trial, one of the computer-controlled ship’s movements responded to the user’s movements in a “hostile” manner. The other five “distractor” ships moved independently of the user. The “hostile” ship would do one of two things—hunt or shadow. Hunting was considered movement designed to eventually catch the user ship. An algorithm computed which directional movement produced the greatest reduction in distance between the two and moved the ship in that direction as the user ship moved. Shadowing aimed to generally keep a consistent distance from the user ship through replication of their movements. For instance, if the user moved left, the shadowing ship also moved left. If the user ship moved toward the shadowing ship, the shadowing ship would move away so the distance between the ships stayed the same. These target movements occurred simultaneously with the user’s movement that triggered it.

The behaviors of the five distractor ships were assigned one of two possible movement patterns. Three of the ships had “targeting” behaviors, meaning they moved toward their own invisible target goal, coded as an invisible point on the coordinate grid. The other two ships exhibited “patrol” behaviors, where they moved in a rectangular course that circumnavigated the center of the screen. Patrolling ships were randomly assigned a patrol pattern, a starting position and direction of travel on each trial.

Three trials were given as practice. The practice trials involved one hunting behavior, one shadowing behavior, and a trial in which the aid was introduced. On the first two practice trials, the hostile ship was displayed with a different color than the distractor ships and the type of hostile behavior was announced before the trial started. This allowed participants to practice working through a scenario but also showed the difference between hostile behaviors. The third practice trial included the aid, which highlighted what it thought to be the likely hostile ship in yellow after five moves (detailed below).

The movements of both the hostile ship and the distractor ships included some uncertainty. On every move of every trial, based on a computer-generated random function, each ship would move according to its intended plan with a 75% probability. Approximately 25% of ships’ movements would be in some arbitrary direction other than the optimal direction for their goal. For example, a ship that was hunting would move closer to the user on approximately 3 out of every 4 moves, with the 4th move taking the ship in a randomly chosen vector direction. Similarly, the distractor ships were programmed to move in an orderly fashion unrelated to the movements of the user’s ship, but about 1 in 4 of their movements would be in a random direction.

The goal for the participant was to move their ship until they could identify the hostile ship, as well as identify the type of hostile behavior: shadowing or hunting. On each trial, the participant was required to make at least five moves, but no more than 35 moves, in whatever pattern they chose before determining which ship they believed was hostile. Once they had collected enough information to make their decision, they clicked on the “End Trial” button, which introduced a question across the top of the screen, asking the participant to select the number of the hostile ship and the type of hostile behavior. The instructions explained that there would always be a hostile ship on each trial. The default response was “None” for the hostile ship number and “Neither” for the type of hostile behavior. This forced the participant to affirmatively select a response. After they selected their response, they then clicked “Submit” and were given feedback if their response was correct, but not the reasons for an incorrect response.

### Decision aid

A decision aid was provided that highlighted one ship in yellow that was ranked as the most likely threat on each trial, starting at the sixth move. The decision aid did not appear until the sixth step because it needed to accumulate evidence on the first few steps to be accurate enough to be useful. This also encouraged participants to collect their own evidence of hostility rather than only relying on the aid. The ranking of ship hostility was determined by a simple algorithm that was dependent on the users’ movements and the movements of the other ships. For hunting, the algorithm counted the number of times each ship moved in the same direction that a hunting ship would have moved. A similar measure was calculated for shadowing behavior such that the number of times each ship moved in the same direction a shadowing ship would have moved was counted.

Therefore, the reliability of the aid in detecting the hostile ship further improved as the number of participant moves within a trial increased. Participants were told in the instructions that the aid became more accurate with more information from steps. After each move, the threat ranking was recalculated, and the appropriate ship was indicated by turning yellow. If there was a tie in the threat rankings, it was resolved by randomly highlighting one of the ships tied for most threatening.

Moreover, reliability of the threat signal was contingent on the user’s moves. For example, if a user started to move in the same direction as a distractor ship that was patrolling, then the threat ranking algorithm might produce a false alarm for that distractor ship. For the user to achieve a high degree of accuracy of threat behavior assessment, they needed to be thoughtful in their choices of movements. Still, through simulations of a participant moving in three simple strategies (always in a straight line, in a circular path, and in a random path) the average accuracy of the simulated aid was found to improve considerably with repeated moves and approached asymptotic accuracy of 94% after approximately 24 moves.

Half of the participants were randomly assigned to the transparency condition, where the highlighting remained the same but participants were provided a statement indicating the reasoning for the highlighting based on behavior such as, “Ship 2 has been shadowing your ship for 13 out of the last 15 moves.” The format of the message remained the same but the ship number, ship behavior, and count of moves could update with each step. This was provided in text below the area of the screen with the moving ships (Fig. 1). The information was presented in this way so that it was easily available for the participant to see, as it was located near the buttons that moved the usership, but also did not create clutter on the screen near the ships. This transparency manipulation is classified as a “medium” level of transparency under the Bhaskara et al. (2020) taxonomy. Providing participants with the reasoning of the aid is the basic form of transparency that has the most consistent benefit to performance.

### Procedure

Each participant was presented with three blocks of trials after a short three trial practice section. Block 1 had eight unaided trials, Block 2 had 16 aided trials, and Block 3 had two unaided trials. Block 1 was treated as baseline performance and a chance for participants to better understand the task of diagnosing hostile behaviors. Block 2, with the aid, had double the trials as Block 1 because these data speak to the core research questions. Block 3 was implemented as a brief final check for practice effects, and to explore how unsupported performance may have differed after the aid was taken away. Performance on the last trial of the Block 2 did differ from the first trial of last block: *V* = 1909.5, *p* < 0.001 and from the second trial of the last block (trial 26) *V* = 1470, *p* < 0.001. So, although the number of trials in Block 3 was low, there was enough power to detect differences from the automation trials that immediately preceded the last block. Participants were instructed that the aid would be withdrawn during these final two trials. Completing all 26 trials took approximately 45 min.

On each trial, all starting positions, movement of computer-controlled ships, and the identity of the hostile ship were pseudo-randomly chosen. In each block, there were equal numbers of trials having hunting or shadowing, although the order of hunting and shadowing trials was randomized in every block. Additionally, participants were randomly divided into two conditions; in one condition they received the transparency manipulation that accompanied the aid within Block 2, making transparency a between-subjects variable.

Finally, after completing all three blocks, participants were asked a series of three survey questions. Participants could rank their answers on a Likert scale from 1 (Not at all) to 7 (Completely). The first question stated, “Did you trust the aid;” the second question stated, “Did you comply with the aid,” and the third question stated, “How confident are you in your own ability to do the task?”.

## Results

### Accuracy

**Fig. 2:**
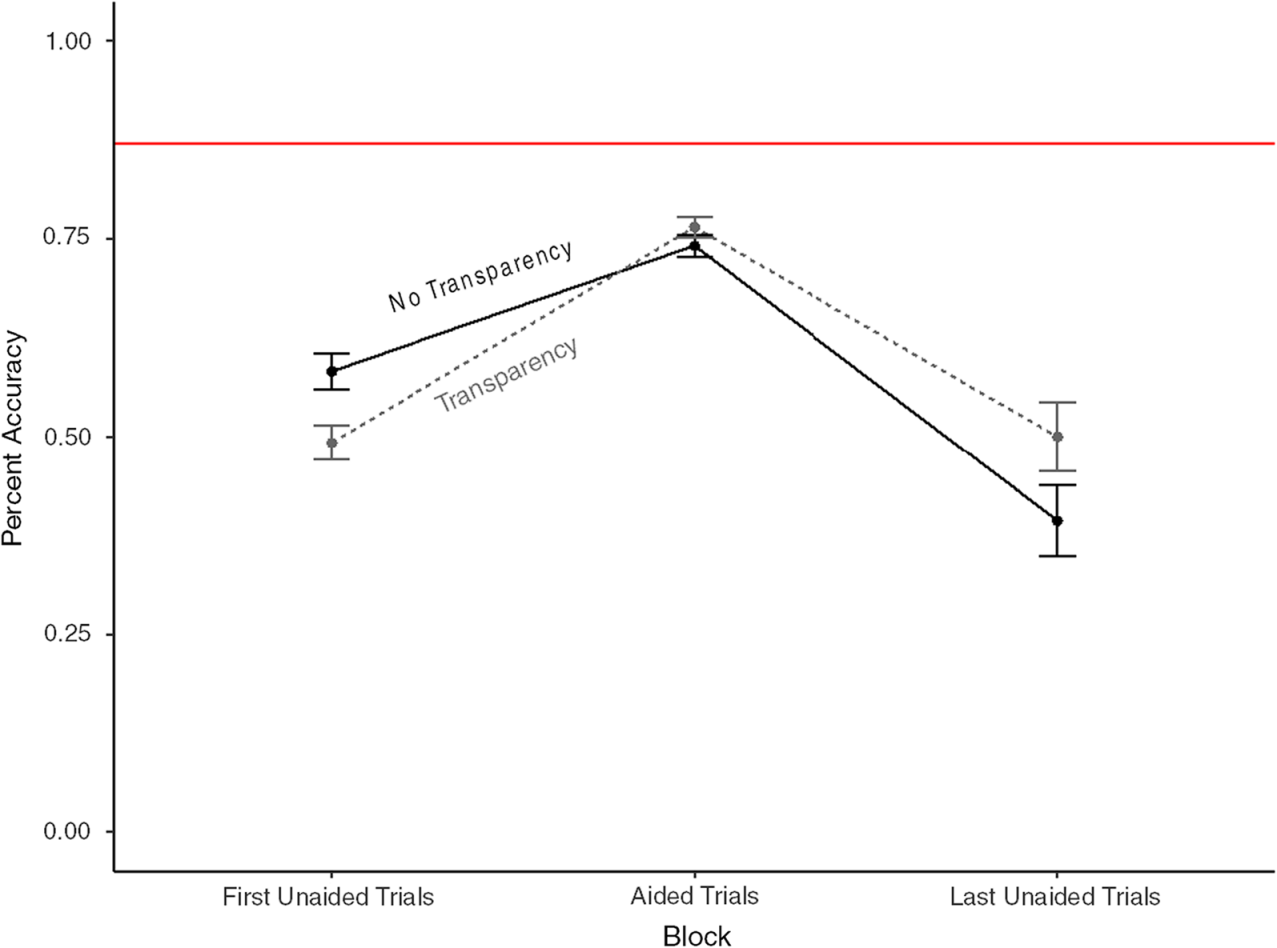
Accuracy in detecting the correct ship across blocks. Error bars represent one standard error of the mean. The red horizontal line indicates the overall 87% mean accuracy of the aid

Figure 2 presents the mean accuracy, operationalized as percentage of correct ship and behavior detection, across the three blocks of trials: without, then with, and then again without the aid. The data in the final block were not normal, as it only consisted of two trials, so a Kruskal–Wallis test was run to determine whether there were differences in accuracy across blocks. It showed that there were differences (H(2) = 196.67, *p* < 0.001), as can be seen in Fig. 2.

Follow-up paired comparisons of the block test were run to test the first hypothesis, that the decision aid would enhance performance in detection of hostile movements. A t-test was run between the first and second blocks and revealed clear assistance offered by the aid (75% accuracy; (*t*(125) = − 11.05, *p* < 0.001, *d* = 1.03). However, the performance in the second block with the aid was lower than the aid’s own accuracy (87%; *t*(125) = − 9.70, *p* < 0.001, *d* = 0.91). The difference between the second and third blocks was also significant with a Wilcoxon signed-rank test (*V* = 6445, *p* < 0.001). Importantly, the decrease in performance from the earlier to the later unaided block was significant (*V* = 3800.5, *p* = 0.01) ruling out the possibility that the improved performance from the initial unaided trials (Block 1) to the aided trials (Block 2) was just an artifact of practice at the task. If it was, participants would have done better, not worse, on Block 3. Additionally, accuracy across the trials within the blocks remained relatively stable, if not with a slight increase, suggesting that the decrease in accuracy in Block 3 was not due to fatigue or vigilance decrements.

A separate t-test was run to investigate whether transparency impacted performance in Block 2. There was no indication of a significant difference (*t*(124) = 0.75, *p* = 0.45, *d* = 0.13), indicating that while the aid improved performance, transparency did not have a differential impact on overall outcomes. The transparency manipulation told participants information about a potentially hostile ship’s behavior, and so as a post hoc exploratory analysis, accuracy was investigated further through the decoupling of detecting which ship and which behavior was exhibited. Overall, when the correct ship was chosen, the correct behavior was also chosen 93% of the time. This stayed true in both the transparency (93.5%) and no-transparency condition (92.9%), despite transparency providing behavior information that could have improved accuracy.

### Trust and compliance

Trust in the automation was subjectively rated on a scale of 1 (not at all) to 7 (completely). Overall, trust was rated at 5.26, with higher trust when there was a transparency explanation (5.49) than without an explanation (5.03; *t*(124) = − 2.08, *p* = 0.03, *d* = 0.37).

*Compliance*. Objective compliance was first measured by the proportion of trials in which the user agreed with the automation’s recommendation, regardless of accuracy. Overall the mean compliance rate was 87% (95% CI [86%, 89%]). However, when looking at compliance across all trials, it is difficult to know whether the participants were blindly agreeing with the aid (automation bias), rather than ignoring the aid and using unaided perceptual information to make the judgment with high accuracy. One insight may be obtained from examining the compliance rate on that specific subset of trials in which automation was wrong. This value was 71% (95% CI [65%, 77%]), a value significantly less than the mean 87% reliability of the aid (*t*(244) =  − 5.38, *p* < 0.001, *d* = 0.34), indicating that participants were not always simply blindly or randomly agreeing with the aid, nor were they just probability matching with its reliability rate.

When looking at compliance rates on automation-wrong trials between the two aided conditions, there was no effect of transparency. Compliance across all trials was very similar at 86% without transparency and 88% with transparency (*t*(124) =  − 1.02, *p* = 0.30, *d* = 0.18). On automation-wrong trials, compliance was 68% without transparency and 74% with transparency (*t*(100) = − 0.85, *p* = 0.39, *d* = 0.17). This suggests that, in contrast to the third hypothesis, transparency did not impact compliance at all, nor was there an impact on automation bias.

### Evidence accumulation

**Fig. 3:**
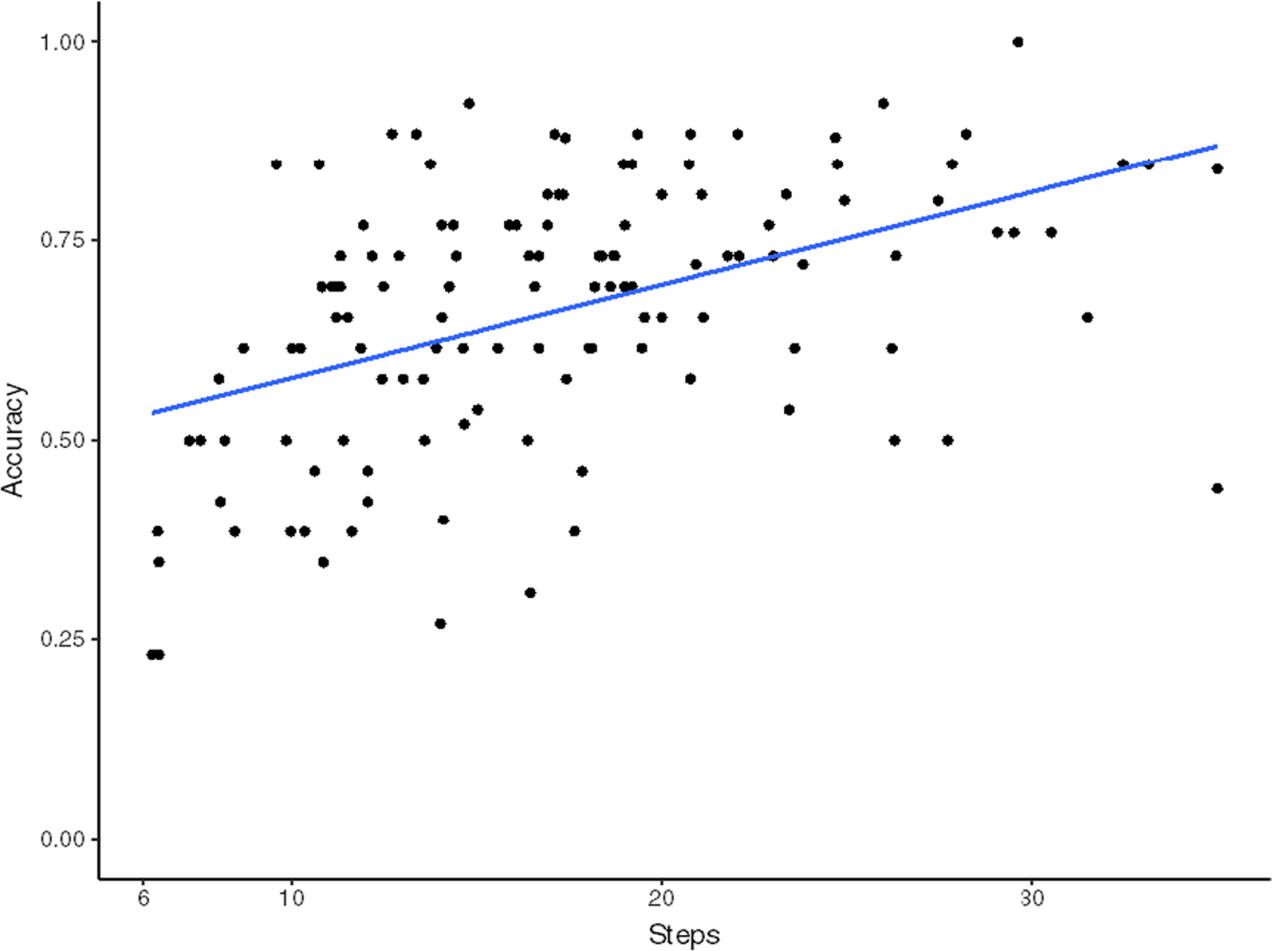
Speed-accuracy trade-off. Each data point represents the average accuracy of a participant across all trials. These data include participants from both conditions

To examine the process of decision making, we examined the speed-accuracy trade-off. Across all trials, participants who took more steps and hence more time increased their accuracy (*r* = 0.47, Fig. 3). This trend was true with (*r* = 0.37) and without automation (*r* = 0.33). Additionally, the average number of steps taken was significantly lower on trials with automation (15.85 steps) than without automation (20.01; *t*(125) = − 11.09, *p* < 0.001, *d* = 0.59). This, combined with the higher accuracy seen on automation trials, indicates that the decision aid allowed users to make correct decisions more rapidly.

There was no difference in the number of steps taken with transparency (16.5) versus without (15.1; *t*(124) = 1.15, *p* = 0.24, *d* = 0.20). This indicates that the additional information from the aid in the transparency condition did not drastically change the information acquisition process that users followed to choose the correct ship. The trend for increasing accuracy with increasing number of steps was consistent in both aided conditions (*r* = 0.40 with transparency and *r* = 0.32 without transparency).

Given that transparency affected neither process (steps taken) nor the product (accuracy of decision outcome), we wanted to assess whether the transparency information was attended at all. To do so, we examined the difference in processing time between steps, between the transparency and the no-transparency condition. Processing time was operationalized by the time spent between steps, after the participant moved their ship on one step and before they moved it on the next ship. Due to multiple comparisons, a Bonferroni correction was implemented, making the new alpha level = 0.01. In the no-transparency condition, there were no differences in time across any of the steps (all *p* > 0.04). In the transparency group, the processing time on the trial when transparency was introduced (*M* = 2.3 s) was significantly longer than the time on the step before (*M* = 1.7 s; *t*(283) = − 4.14, *p* < 0.001, *d* = 0.49), which can be assumed to relate to the time taken to read the information. Such a delay was not observed on the prior step in the no-transparency condition (*M* = 2 s), before automation was introduced (*t*(143) = 2.19, *p* = 0.02, *d* = 0.23). Hence, although the initial presentation of transparency showed signs of additional processing of that information, the combined other analyses suggest this knowledge subsequently made little substantive impact on the decision processes themselves.

### Removal of the aid

The final way in which the effect of transparency was investigated was to assess whether there were any differences that may exist on the final two trials when the automation was removed. There was no difference in the number of steps used on the final two trials, with both groups using approximately 17 steps (*t*(250) = − 0.35, *p* = 0.72, *d* = 0.04). Accuracy on the last two trials for the transparency group was nominally higher (50%) than the no-transparency group (39%) although this difference was not significant with a Wilcox signed-rank test (*W* = 1683.5, *p* = 0.11).

## Discussion

This study set out to examine how, in the context of dynamic decision making, an automated decision aid could assist the diagnostic performance of the human; furthermore, we examined how transparency of an automated aid impacts both the decision-making process and the final decision outcome. While transparency of diagnostic aids has been frequently examined in static decision contexts (i.e., Kunze et al., 2019; Odour & Wiebe, 2008; Mercado et al., 2016), it does not appear to have been examined in the context of evidence accumulation for dynamic decision making. Therefore, the current study explored whether transparency impacts the decision-making process and whether the trends seen in static decision making with automation remain the same in dynamic decision making. One possible source for differences would be increased workload influencing the ability of human operators to process and use the transparency information. Four main hypotheses were posed.

The first hypothesis was that the aid would improve performance, and it did by approximately 25%, thereby confirming the first hypothesis. This finding was consistent with much of the prior research on human–automation diagnosis teaming (e.g., de Visser & Parasuraman, 2011; Hutchinson et al., 2022; Wright et al., 2018), although little has been performed in dynamic decision making (Kersthold & Raaijmakers, 1997). Furthermore, improvements are found to be particularly large when there is a large disparity between the aid’s performance and that of the unaided human (Boskemper et al., 2021), as was the case here. Yet, given the aid’s high reliability (87%), the performance of the human–automation team was considerably less than optimal, as shown in Fig. 2, and certainly did not demonstrate any synergy such that the team would perform better than either its human or automation component. These findings are consistent with much prior research on human interaction with automated diagnostic systems in static decision-making contexts (Bartlett & McCarley, 2017; Goddard et al., 2014; Wiczorek & Meyer, 2014), although the current results uniquely demonstrate that this shortfall applies to dynamic decision making as well. Indeed, the challenge implied for increasing calibrated compliance with a decision aid may be even greater for dynamic decision-making situations, where the underlying workload leaves limited scope to obtain an appreciation for how the automation is functioning. We return to this issue below in the discussion of the outcomes of transparency manipulations.

In the current results, the aid was found to increase performance in a way that was not totally accomplished by blind adherence to its advice (i.e., the automation bias). This conclusion reflected the fact that the compliance rate on automation wrong trials was less than the overall automation reliability, indicating that several times the human overrode and contradicted the automation assessment. It is important to note that in a similar classification paradigm, using more formal modeling techniques, Strickland et al. (2023) also observed what can be considered a mixture of the two strategies: blind adherence and more deliberative contradiction.

Of interest within the dynamic context was the extent to which the aid might have helped participants become better at the task when the aid was withdrawn (Hypothesis 2). Specifically, with the aid generally being more accurate than the human early on in each trial, this would allow participants to watch one ship for a period of time and learn to better identify the hostile behavior, which may have transferred once the aid was removed. Had this been the case, there would have been improvement on Block 3 when the aid was withdrawn, relative to Block 1. This was not the case, and instead there was a significant worsening of performance, a phenomenon similar to the “automation gone” effect observed by Wickens et al. (2015). Thus, it would appear that even when the aid was present and used, the working memory demands of the task were sufficiently high that participants did not allocate resources to improve their skill at diagnosis. In the parlance of cognitive load theory (Sweller, 1994), we would argue here that the intrinsic load of this task was sufficiently high so as to prevent cognitive load from being reallocated to the germane load demands of skill acquisition.

The third hypothesis stated that transparency would improve performance, specifically because the additional information provided participants with information on the behavior of the hostile ship. This was not supported, with no difference between the transparency and no-transparency conditions. This was unexpected because although the literature has indicated that sometimes transparency does not improve performance (Göritzlehner et al., 2014; Loft et al., 2021; Zhang et al., 2022), typically reasoning-transparency (used here) does improve performance (Bhaskara et al., 2020; Pharmer et al., 2022; van de Merwe et al., 2022). We also anticipated that transparency would increase trust in the aid. This was confirmed, but it was apparent that such a trust increase did not produce any increase in reliance toward the more calibrated level of 87% (the aid reliability) in a way that would benefit performance. As observed elsewhere (e.g., Hutchinson et al., 2022; Pharmer et al., 2021, 2022; Strickland et al., 2023) and proposed by Lee and See (2004), trust and reliance or dependence are far from closely coupled.

While the latency analysis revealed that the transparency information was at least processed, the lack of improvement in accuracy with transparency may be because the accuracy of the identifying the hostile ship and diagnosing the hostile behavior are closely coupled. Post hoc analyses showed that when the correct ship was indicated, the correct behavior was also chosen 93% of the time. Perhaps transparency, in this case, was not any more helpful than automation alone because participants were able to identify the behavior quite accurately once attention was directed to a candidate entity. More specifically, if humans could identify a single ship’s behavior at the same level or more accurately than the automation, then the additional information about a ship’s behavior would not improve performance. One potential reason in this case is that the movements selected by the human did not provide adequate diagnostic value to the algorithm underlying the automation. Another possible explanation is that the high working memory demands of dynamic decision making (keeping track of past moves as evidence is accumulated) competed for cognitive resources with the task of reading, using, and interpreting the reasoning information that constituted the transparency manipulation. Such a cognitive load explanation has been offered by Zhang et al (2022) to account for their failure to observe a benefit for transparency in a decision aid.

Fourth, it was hypothesized that transparency would change the way users made decisions. While a directional hypothesis was not specifically posed, differences in compliance rates, steps used, and accuracy when the aid was removed were expected. However, none of these dependent variables revealed significant differences when transparency was added. Accuracy on the final two trials, when the aid was removed, showed a trend toward increased accuracy in the transparency condition, but it was not significant (*p* = 0.11). However, differences did appear in the time between steps in the transparency condition at the first onset of the decision aid information, with no difference in the no-transparency condition. This suggests that participants did see the information provided and some likely considered it in their reasoning. Combined with the null results in other aspects and the post hoc finding of the close coupling of correct ship and behavior detection, our findings suggest that the lack of a benefit of transparency here was not due to ignoring the information. This would suggest that the human operators either found the information provided was not the most useful in this context, or were unable to integrate into their decision-making cycles because of the high working memory load of the task. These findings support the conclusions found in Skraanging and Jamieson (2021), who suggested that transparency manipulations must be specialized to the context in a way that provides necessary information.

While transparency was not beneficial in this context, conclusions can still be inferred about its impact on the decision-making process. It is likely that to provide a benefit, transparency must provide information that is used in the process of making a final decision, whether that be through augmentation of the raw data or the processing of the automation. Here, post hoc analyses indicated that information about a ship’s behavior may not have been beneficial to the users in their accuracy, but it also did not improve their ability to use the automation at an appropriate level. This suggests that it is not just that the close coupling of behavior and ship identification is responsible for the lack of a benefit. Instead, the benefit to performance came only from the automation highlighting a single ship, even though previous literature suggested that decreasing set size improved performance less than the automation did here (Patton et al., 2022). This seems to suggest that something about the automation itself changed the way users completed this task.

Overall, these findings suggest that the load from the underlying task itself in a dynamic decision-making context can have significant impacts on interactions with automated decision support. Ultimately, the deleterious effects of that cognitive load may require a greater level of exposure both for learning to use and integrate the automation into the decision cycle and for developing an appreciation of its capabilities and limitations.

### Limitations

The limitations of the transparency manipulation are important to consider. First, the transparency text was located in such a place that required some effort to access. While speculative, the effort required to repeatedly access the information may have been deemed not worth the value of the information by participants and therefore was largely ignored after the initial presentation.

Additionally, although the current paradigm served to investigate automation in a dynamic decision-making context and not a way to investigate exact questions of maritime intent detection, the simplicity lends itself to a few other limitations. Although the paradigm mimics real Naval displays and tasks, participants were naïve adults without Naval experience. This may limit the generalizability of the results. The use of only two behaviors, one of which was always present, also leaves open questions about generalizability to other types of suspicious movement behaviors and less certain situations.

## Conclusions

The current study set out to investigate how transparency and an automated aid impact human–automation team performance in a dynamic decision environment. Consistent with much of the prior research in static decision-making tasks, the presence of the automation improved performance relative to the human-only level, but performance of the human–automation team was lower than the automation-only performance level. Then, when the aid was abruptly withdrawn, participants showed little evidence that they had learned how to do the task manually, by availing their residual attention during the trials when the aid was present. This, in conjunction with the accuracy levels and compliance rates, suggests that the improvement to performance seen on trials with the aid is largely a result of the aid’s accuracy and not the human working in conjunction with the aid in order to improve their own judgment skills.

Transparency made no significant impact on any metric of performance in this study. This is a novel finding because this paradigm was set in a dynamic decision-making environment, which is in contrast to most automation and transparency research that is set in a static decision environment. These results suggest that transparency may not be as useful in a dynamic environment, particularly with high cognitive demands. At least in the current context, and potentially more generally for dynamic decision making, the current findings suggest that the general prescription from Bhaskara et al. (2020)—that providing reasoning from the automated system can be beneficial because it serves to increase transparency—did not hold. Although more research is needed to replicate this finding and investigate explanations, it may be that when human operators are collecting evidence themselves in conjunction with an automated aid, the additional information from transparency is not as useful as in static decision making, where the human may not have insight into how the aid has made its decision.

## Data Availability

The datasets used and/or analyzed during the current study are available from the corresponding author on reasonable request.
